# From a novel pathogenic *SAMD9L* variant to cohort‐wide insights: Whole‐genome sequencing highlights somatic genetic rescue and phenotypic heterogeneity

**DOI:** 10.1111/bjh.70563

**Published:** 2026-05-19

**Authors:** Hadjer Dellal, Roman Klifa, Lise Larcher, Marie Passet, Tristan Celse, Laetitia Gouas, Maud Tusseau, AURAGEN Consortium, Alexis Praga, Virginie Bernard, Mathieu Fusaro

**Affiliations:** ^1^ Immunology Department Laboratory, Referral Medical Biology Laboratory University Hospital of Toulouse Toulouse France; ^2^ Pediatric Intensive Care Unit Hôpital Universitaire Pellegrin, Université de Bordeaux Bordeaux France; ^3^ Department of Biological Hematology Saint‐Louis Hospital, INSERM U1342, Saint‐Louis Research Institute and Paris Cité University Paris France; ^4^ Service de Génétique, Génomique et Procréation, Hôpital Couple‐Enfant, CHU Grenoble Alpes Université Grenoble‐Alpes La Tronche France; ^5^ GCS AURAGEN Lyon France; ^6^ Cytogénétique Médicale Centre Hospitalier Universitaire de Clermont‐Ferrand, CHU Estaing, INSERM U1240 Imagerie Moléculaire et Stratégies Théranostiques, Université Clermont Auvergne Clermont‐Ferrand France; ^7^ Centre International de Recherche en Infectiologie, Inserm, U1111 University Claude Bernard, Lyon 1, UMR5308, ENS de Lyon Lyon France; ^8^ Department of Medical Genetics, Hospices Civils de Lyon Hospices Civils de Lyon Lyon France; ^9^ Immunology Department Laboratory, Referral Medical Biology Laboratory, Toulouse University Hospital Center, INFINITy, Toulouse Institute for Infectious and Inflammatory Diseases, Inserm U1291, CNRS U5051 University Toulouse III Toulouse France

**Keywords:** bone marrow failure, clonal evolution, *SAMD9*, *SAMD9L*, somatic genetic rescue (UPD7q), trio whole‐genome sequencing

## Abstract

Germline gain‐of‐function variants in sterile alpha motif domain–containing 9‐like (*SAMD9L*), located on chromosome 7q, cause a multisystem disorder characterized by bone marrow failure, immunodeficiency and variable neurological involvement. Disease evolution is frequently shaped by somatic genetic rescue (SGR), most commonly through monosomy 7, somatic loss‐of‐function (LOF) variants in *cis* or uniparental disomy of 7q (UPD7q). We report a 6‐month‐old girl presenting with pancytopenia and severe infections, in whom we identified a novel heterozygous *SAMD9L* variant. Trio‐based whole‐genome sequencing demonstrated maternal transmission and revealed distinct SGR mechanisms in both the proband and her asymptomatic mother, including UPD7q and an additional somatic LOF variant. Longitudinal molecular follow‐up showed dynamic clonal evolution associated with haematological improvement. To assess the broader relevance of *SAMD9/SAMD9L* variants, we also performed a gene‐centred analysis of the 2025 French Genomic Medicine Initiative (PFMG2025), enabling the identification and classification of additional candidate variants. These findings support systematic screening for SGR, including mosaic UPD7q, even in cases with apparently normal variant allele frequency, and highlight the importance of integrating haematopoietic and non‐haematopoietic tissues in diagnostic workflows for inherited bone marrow failure syndromes.

## INTRODUCTION

Sterile alpha motif domain–containing 9‐like (*SAMD9L*) is a ubiquitously expressed cytosolic protein located on chromosome 7q21 and belonging to the STAND family of growth‐restricting proteins.^1^ Together with its paralogue *SAMD9*, it contributes to antiviral defence,^2^ regulation of cell proliferation^3^, ^4^, ^5^ and inhibits translation.^6^, ^7^, ^8^ Gain‐of‐function (GOF) germline variants in *SAMD9L* or *SAMD9* were discovered in 2016 as the cause of multisystem disorders with autosomal dominant inheritance and variable penetrance.^9^ Indeed, most pathogenic *SAMD9L* variants are heterozygous missense (>90%) variations that enhance the protein's intrinsic growth inhibitory activity. The first patients described with such variants in *SAMD9L* or *SAMD9* were characterized as ataxia–pancytopenia syndrome^10^ or MIRAGE syndrome, ^11^ (an acronym for myelodysplasia, infection, restriction of growth, adrenal hypoplasia, genital phenotypes and enteropathy), respectively. Since these initial descriptions, the phenotypic spectrum of SAMD9/SAMD9L disorders has expanded, with haematological dysfunction as a common feature.^9^ Thus, *SAMD9/9L* syndromes represent 8%–18.6% of bone marrow failure and myelodysplastic syndrome cohorts with paediatric onset.^12^

Remarkably, nearly two‐thirds of affected individuals develop somatic genetic rescue (SGR) contributing to haematopoiesis correction.^12^ SGR is a post‐meiotic mechanism through which the pathogenic germline allele is modified or lost, typically resulting in phenotypic correction.^13^, ^14^ Three main SGR mechanisms have been described: (1) Uniparental disomy of 7q (UPD7q), which duplicates the wild‐type allele and can lead to long‐term haematological normalization; (2) somatic *SAMD9L* loss‐of‐function (LOF) variants in *cis* with the germline GOF variant, partially reversing the growth restriction; (3) Monosomy 7, the latter being associated with leukaemic risk. These mechanisms contribute to the highly variable phenotypes observed within families and between individuals carrying the same germline *SAMD9L* variant.

Here, we report a 5‐month‐old girl presenting severe opportunistic infections, which evolve towards pancytopenia, in whom we identified a novel heterozygous germline *SAMD9L* variant with a presumed GOF effect. Trio‐based whole‐genome sequencing (WGS) demonstrated maternal transmission of this variant. Remarkably, the asymptomatic mother harboured two independent SGR events: a somatic LOF *SAMD9L* variant and a mosaic segmental UPD7q (sUPD7q). The proband also displayed early signs of UPD7q‐driven rescue. This family illustrates the dynamic interplay between germline *SAMD9L* variants and compensatory somatic events, highlights intergenerational variability in penetrance and supports the use of WGS to classify this newly identified *SAMD9L* variant as pathogenic.

In addition, to evaluate the broader prevalence and potential underdiagnosis of *SAMD9*/*SAMD9L*‐related disorders, we performed a gene‐centred interrogation of the 2025 French Genomic Medicine Initiative (PFMG2025) dataset, which gathered 11 913 genomes from 60 rare disease categories. This complementary approach allowed systematic screening for pathogenic or candidate variants within these genes across a large cohort of unsolved rare disease cases.

## MATERIALS AND METHODS

### Whole genome sequencing

Whole genome sequencing was performed following the recommendations of France Genomic Medicine Plan. Whole blood extracted genomic deoxyribonucleic acid (DNA) was sequenced according to standard procedures for a polymerase chain reaction (PCR)‐Free genome on a NovaSeq6000 instrument (Illumina). In general, genome sequencing was achieved with a median target coverage of 30×. Sequencing data were aligned to the GRCh38p13 full assembly using BWA 0.7+. Variants were called by several algorithms including GATK4+, Bcftools1.10+, Manta1.6+, CNVnator0.4+ and annotated using variant effect predictor VEP 98.3 and resource gencode 32. Detected variants were prioritized using in‐house procedures. Further details are available on request on http://www.auragen.fr. Detection of mosaic uniparental disomy was performed using the Baracuda tool developed within the AURAGEN platform (see Supporting Information for details).

### NGS panel sequencing

A myeloid neoplasia gene panel was routinely analysed by capture‐based next‐generation sequencing (NGS) including a minimal set of 108 genes (see Supporting Information for full list of genes). Preparation of the libraries, bioinformatics pipeline for identification and interpretation of punctual mutations and copy number aberrations were carried out as previously described.^15^

### Database transversal analysis of *SAMD9* and *SAMD9L*

Whole‐genome sequencing data generated within the PFMG2025 national rare disease programme were retrospectively screened to identify rare variants in *SAMD9* and *SAMD9L* across a cohort of patients sequenced for one of 60 rare disease indications. Variant calling was performed using standardized pipelines implemented by the PFMG2025 platform (see Supporting Information for variant filtering strategy). Variants were classified according to ACMG guidelines in the context of the overall genotype–phenotype correlation.

## RESULTS

### Clinical case report

A 5‐month‐old infant presented with severe opportunistic infections, including *Pneumocystis jirovecii* pneumonia and systemic cytomegalovirus (CMV) infection, associated with B‐cell and Natural Killer (NK)‐cell lymphopenia and T‐cell functional impairment despite normal immunoglobulin levels, suggesting an underlying cellular immunodeficiency (Figure S1 and Table S1). She subsequently developed pancytopenia (Table S2). Bone marrow examination showed mild dysplastic features without cytogenetic abnormalities.

Targeted sequencing on bone marrow aspirate identified a *SAMD9L* missense variant (NM_152703.5: c.4529G>C; p.Gly1510Ala) with a variant allele frequency (VAF) of 24%, raising the possibility of somatic mosaicism or germline origin. The variant was initially classified as a variant of uncertain significance (VUS). Overall, this case raised the suspicion of a *SAMD9L*‐related disorder, prompting further genetic and longitudinal analyses (see Supporting Information for detailed clinical, immunological and haematological data).

### Genetic exploration

**FIGURE 1:**
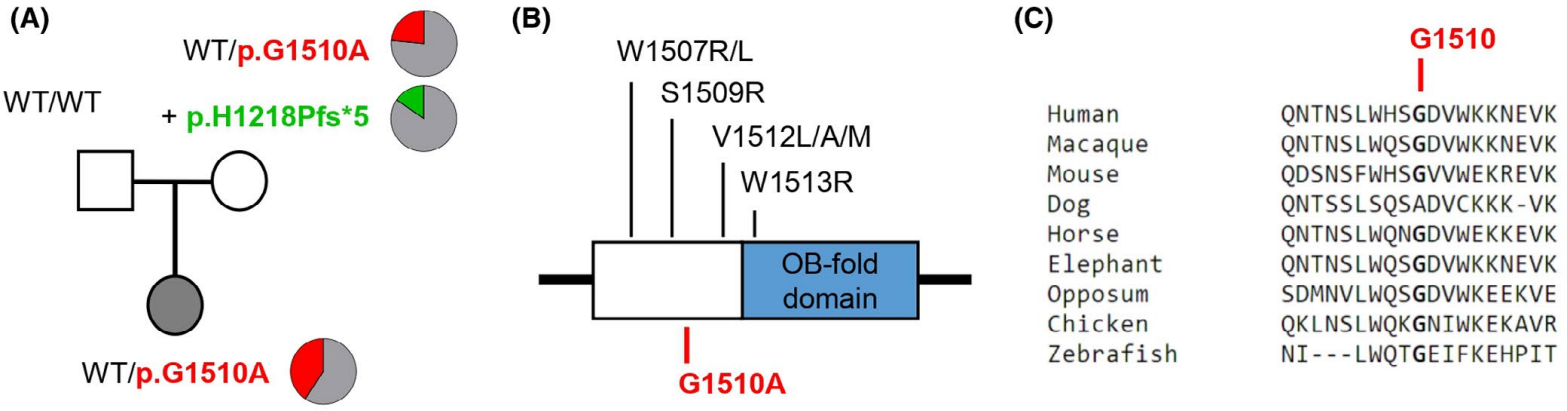
Whole‐genome sequencing identified a novel missense variant in *SAMD9L*. (A) Family pedigree. Segregation of the *SAMD9L* c.4529G>C (p.G1510A) variant (red) and the frameshift p.H1218Pfs*5 second‐site variant (green). Pie charts indicate variant allele frequency (VAF) in blood. (B) Mutational landscape of germline pathogenic *SAMD9L* variants located in the C‐terminal domain (aa 1505–1515). Data are based on variants reported in Sahoo et al. The new variant is shown in red. OB, Oligonucleotide/oligosaccharide binding. (C) Multispecies evolutionary conservation of the amino acid residue G1510. The sequence alignment was performed with Clustal Omega (http://www.ebi.ac.uk/Tools/msa/clustalo/). [Colour figure can be viewed at wileyonlinelibrary.com]

Importantly, prior to the onset of pancytopenia, trio WGS had already been initiated through the 2025 French Genomic Medicine Initiative (PFMG2025), a national plan proposing trio genome sequencing in several rare diseases.^16^ Peripheral blood samples from the proband and her asymptomatic parents had therefore been submitted for analysis within the initial indication of suspected inborn error of immunity (IEI). As expected, the *SAMD9L* variant previously identified in the bone marrow aspirate was confirmed in the patient's blood. The VAF was higher in blood (40.7%, 22/54 reads) than in bone marrow (24%), prompting further investigation. Segregation analysis demonstrated that the variant was maternally inherited, although the maternal VAF was only 23.4% (11/47 reads), suggesting mosaicism. Nonetheless, its presence in the mother strongly supported a germline origin of the variant in the child (Figure 1A). Germline status was definitively confirmed by detecting the variant in DNA extracted from skin fibroblasts. This p.Gly1510Ala variant has not been previously reported in *SAMD9L*‐related disorders nor listed in ClinVar and is absent from both gnomAD v4 genome and exome databases. The Gly1510 residue is located in the C terminal domain of the protein (Figure 1B) and is highly conserved across species, with the exception of a conservative substitution to alanine in dogs (Figure 1C).

### Discovery of somatic genetic rescue mechanisms

**FIGURE 2:**
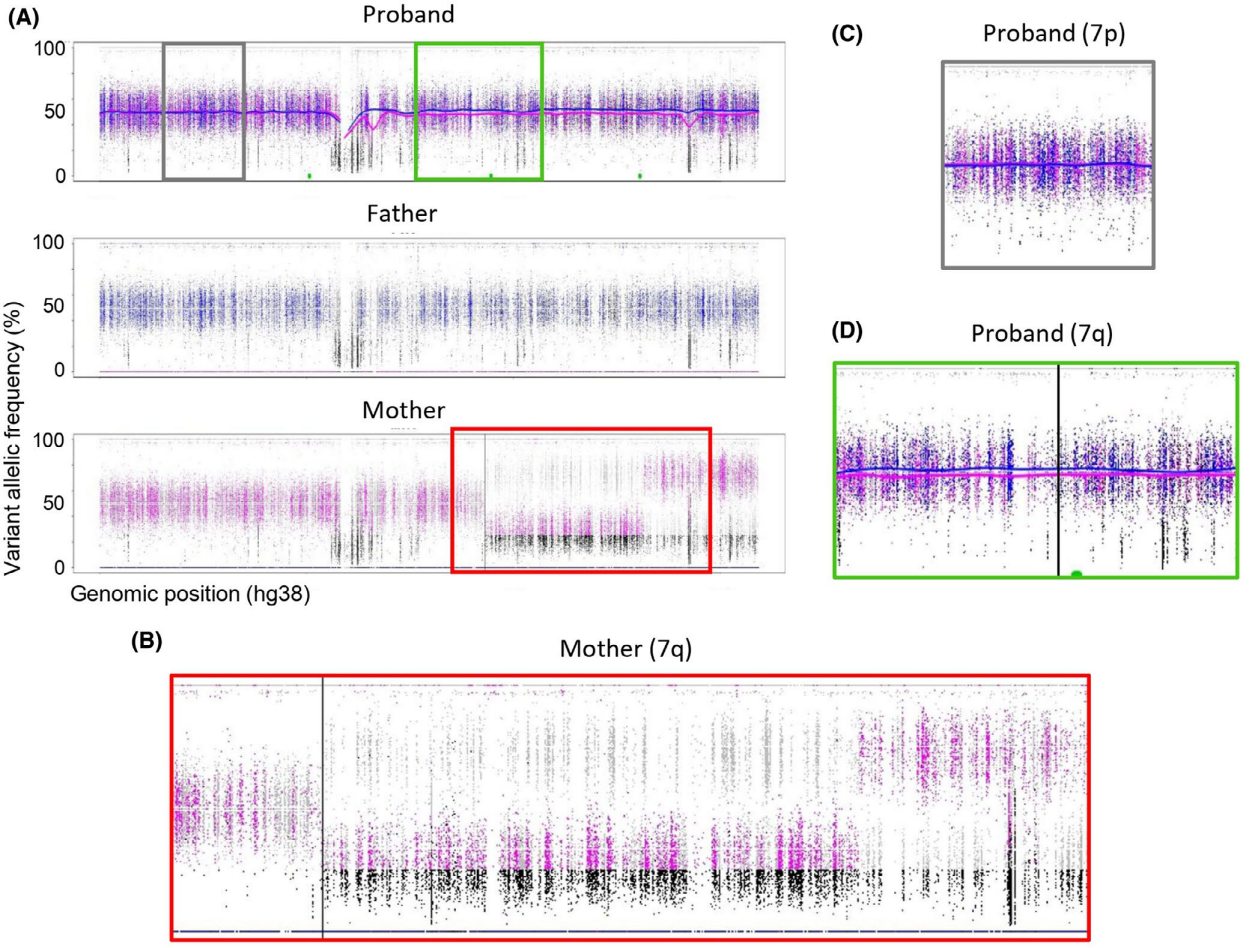
Whole‐genome SNP‐array analysis identified uniparental disomy in both the mother and the proband's blood samples. (A) Genome‐wide representation of all variants on chromosome 7 displayed in an SNP‐array–like plot. The *y*‐axis represents the allele frequency (AF) of the alternate allele for all SNVs, which approximates the B‐allele frequency (BAF) and enables the detection of allelic imbalance and loss of heterozygosity across the genome. Blue dots indicate variants inherited from the father, pink dots indicate variants inherited from the mother and grey dots indicate non‐inherited variants. In the proband, blue and pink lines represent the moving average of AF. Black vertical lines indicate the position of the *SAMD9L* gene. (B) Magnified view of the region highlighted in red in (A), showing a mosaic segmental uniparental disomy (UPD) of chromosome 7q. This somatic event also suggests the occurrence of a crossover. (C, D) Comparison of the moving average of VAFs for father‐ and mother‐inherited variants with magnified view of the regions highlighted in grey (C) and in green (D). (C) Perfect alignment of paternal and maternal VAFs along chromosome 7p. (D) A shift between paternal and maternal VAFs along chromosome 7q, consistent with a segmental UPD with very low‐level mosaicism. [Colour figure can be viewed at wileyonlinelibrary.com]

To understand the discrepancy between mother's genotype and phenotype, we investigated the possibility of SGR. We performed an unbiased search for additional *SAMD9* or *SAMD9L* variants, without applying VAF or transmission filters. This analysis identified a second *SAMD9L* frameshift variant (NM_152703.5: c.3650dup, p.His1218Profs*5) in the mother (Figure 1A), present at a low VAF of 15.4% (6/39 reads), consistent with a somatic event, probably occurring in *cis* with the germline variant. However, this finding alone did not account for the reduced VAF of the germline variant. A novel tool to detect mosaic disomy from WGS data in Auragen called Baracuda was used, based on a single nucleotide polymorphism (SNP)‐array‐like approach (Figure 2A). The mother exhibited a mosaic sUPD7q, with a breakpoint between 7q21.2 and 7q21.3 that contains the *SAMD9L* locus. The shift in the maternally transmitted variants (in pink) from a VAF of 25%–75% was suggestive of a crossover event, which may complicate interpretation (Figure 2B). Importantly, the absence of any decrease in sequencing coverage across this region further supports a copy‐neutral event rather than a mosaic interstitial deletion (Figure S2). Given the frequency of UPD7q in these disorders, we carefully reviewed the proband's SNP‐array profile, despite no abnormality detected by Baracuda. A subtle shift in allelic ratios was detected across 7q (Figure 2D) but not 7p (Figure 2C), consistent with a low‐level mosaic UPD7q. This could explain the intermediate VAF of 40%, slightly below the expected 50% for a heterozygous germline variant without rescue.

### Evolution of somatic genetic rescue and clinical course

**FIGURE 3:**
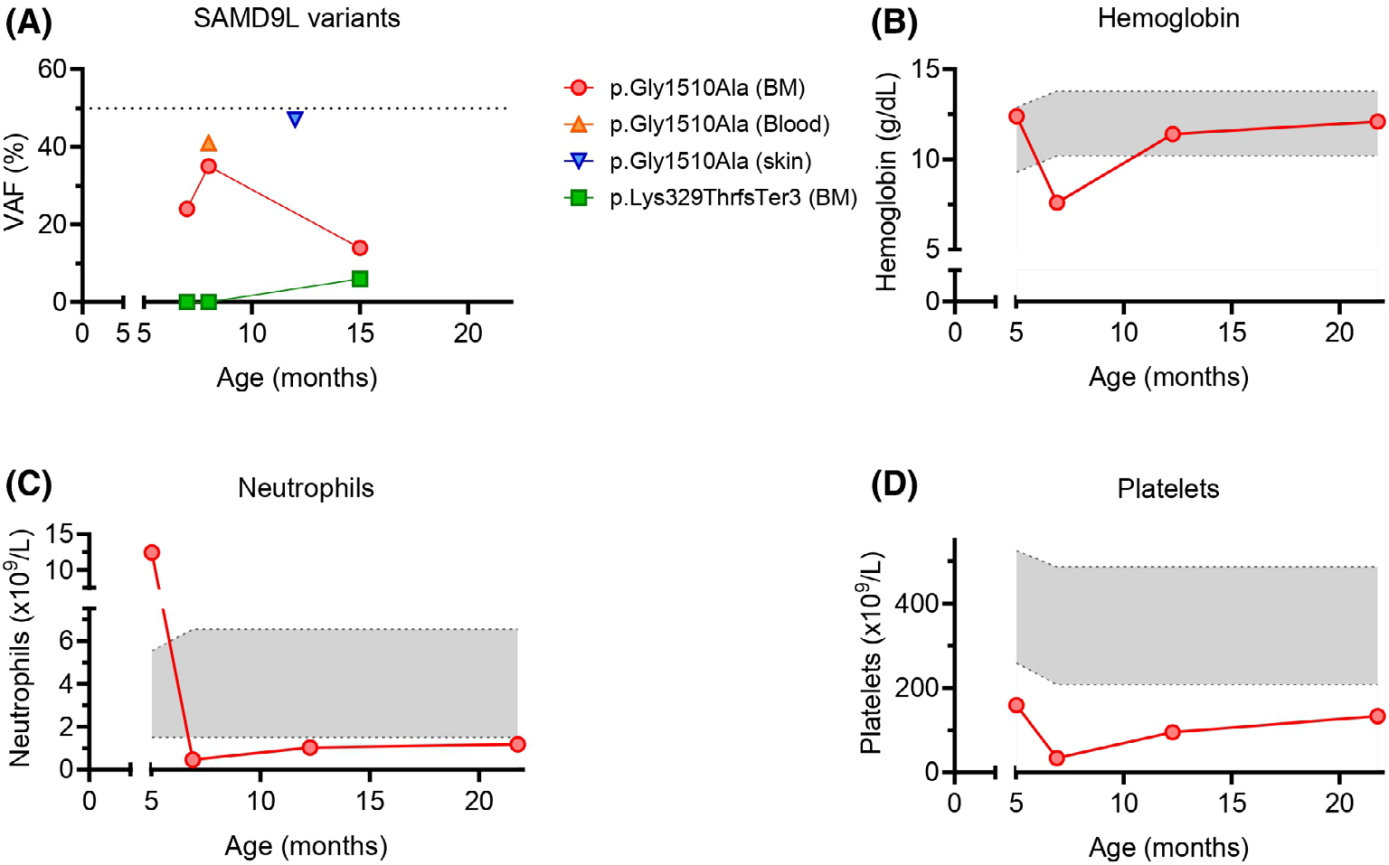
Favourable biological and genetic clonal evolution driven by somatic rescue mechanisms. (A) Longitudinal evolution of variant allele frequencies (VAFs). Changes in the VAFs of the *SAMD9L* variants p.Gly1510Ala and p.Lys329ThrfsTer3 in the proband across different tissues (peripheral blood, bone marrow and skin) from 5 to 15 months of age. (B–D) Longitudinal peripheral blood counts from 5 to 21 months of age, including haemoglobin levels (B), absolute neutrophil counts (C) and platelet counts (D). [Colour figure can be viewed at wileyonlinelibrary.com]

The presence of SGR events, particularly UPD7q, is considered a favourable prognostic factor in *SAMD9/SAMD9L* syndromes, as it reduces the risk of myelodysplastic progression and leukaemic transformation. Therefore, haematopoietic stem cell transplantation was not pursued as initially planned. A conservative watch‐and‐wait strategy was adopted consisting of serial haematological monitoring and repeated high‐coverage NGS sequencing of *SAMD9L* in bone marrow's DNA. A second *SAMD9L* analysis performed on bone marrow, in parallel with WGS, showed a VAF of 35%, slightly higher than the initial result (24%) and approaching the VAF observed in peripheral blood DNA (Figure 3A). Eight months later, follow‐up sequencing demonstrated a marked decrease in VAF, dropping to 14%, consistent with clonal expansion of the UPD7q‐rescued population. In addition, a new somatic LOF *SAMD9L* variant (NM_152703.5: c.985_986insC; p.Lys329Thrfs*3) was detected with a VAF of 6%, potentially representing the emergence of an additional SGR mechanism. These genetic changes correlated with a progressive improvement in haematological parameters (Figure 3B–D).

### Dataset‐wide search for *SAMD9/SAMD9L* rare variants

**FIGURE 4:**
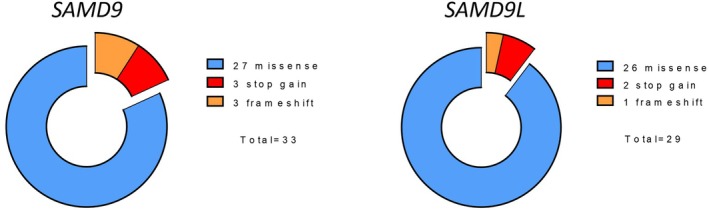
Trio‐based whole‐genome sequencing reveals several SAMD9/SAMD9L variants. Proportion of mutational subtypes of germline SAMD9 and SAMD9L variations identified in the PFMG2025 rare disease sequencing programme using the AURAmatcher tool. [Colour figure can be viewed at wileyonlinelibrary.com]

Given the marked phenotypic variability and the high frequency of SGR in *SAMD9/SAMD9L* syndromes, we sought to determine whether pathogenic coding variants in these genes might be present in patients enrolled in the PFMG2025 rare disease sequencing programme, with a focus on those with sequencing performed by the AURAGEN platform (Lyon, France). To explore this possibility, we used the AURAmatcher tool, which enables gene‐centred queries across the entire dataset of sequenced individuals (11 913 patients from 60 rare disease categories). After excluding variants with minor allelic frequency >0.005% within the general population (gnomAD v4.1), we detected many rare variants in *SAMD9* (*n* = 33) and *SAMD9L* (*n* = 29). The vast majority of those were missense and classified as VUS (Figure 4, Figures S3 and S4). To increase the probability of pathogenic variants discovery, we decided to focus on de novo variations and/or already reported variations and to search for SGR mechanisms.

#### One novel pathogenic *SAMD9* variant

Among the four *SAMD9* variants identified, only one de novo missense variant was clearly associated with a phenotype consistent with the MIRAGE spectrum, in the absence of detectable SGR (Table 1). This previously unreported variant (c.1183G>T; p.Val395Phe), identified in patient P1, was classified as probably pathogenic based on its de novo occurrence and the strong concordance between the clinical presentation and known *SAMD9‐*associated phenotypes.

**TABLE 1:** *SAMD9/SAMD9L* variants identified in PFMG2025 rare disease sequencing programme (WGS on peripheral blood).

Patient	Age (at WGS)	Gene	Variant (HGVS)	Protein	VAF (%)	Inheritance	Prior reports	Genetic rescue	Main clinical indication	PFMG2025 disease category	Phenotypic compatibility	ACMG classification
P1	2	*SAMD9*	c.1183G>T	p.Val395Phe	44	De novo	Not reported	None detected	Primary immunodeficiency. Anaemia. IUGR. enteropathy	Primary immunodeficiency	High	4
P2	3	*SAMD9*	c.4598G>A	p.Arg1533Gln	54	De novo	Reported^17^	Possible germline rescue (co‐occurring pLOF)	Syndromic neuro‐developmental disorder	Developmental and dysmorphic syndromes without intellectual disability	Partial/atypical	5
			c.1030C>T	p.Arg344Ter	54	De novo	Not reported	—			—	—
P3	5	*SAMD9*	c.2048T>G	p.Phe683Cys	26	De novo	ClinVar only (classified as VUS)	None detected	Intellectual disability	Intellectual disability	Low	3
P4	4	*SAMD9*	c.4460A>G	p.Lys1487Arg	58	Inherited (both parents are heterozygous)	Reported^18^	None detected	Intellectual disability. Epilepsy	Intellectual disability	Low	3
P5	13	*SAMD9L*	c.4648T>C	p.Ser1550Pro	48	De novo	Reported^19^	Mosaic UPD7q + somatic pLOF variation	Leukodystrophy. Pyramidal syndrome. Pancytopenia	Leukodystrophy	High	5
			c.2566_2569del	p.Ala856Ter	15	De novo	Not reported	—			—	—
P6	59	*SAMD9L*	c.2807C>T	p.Thr936Ile	52	Unknown	Reported^20^	None detected	Hereditary spastic paraplegia	Early‐onset hereditary spastic paraplegias	Low/atypical	3
P7	5	*SAMD9L*	c.2519T>C	p.Met840Thr	50	Inherited from mother	p.Met840Lys reported^19^	Possible germline rescue (co‐occurring missense)	Intellectual disability	Intellectual disability	Low	3
			c.4231C>G	p.Arg1411Gly	49	Inherited from mother	Not reported	—			—	3

Abbreviations: ACMG, American College of Medical Genetics and Genomics; HGVS, Human Genome Variation Society; IUGR, intrauterine growth retardation; pLOF, predicted loss‐of‐function; UPD7q, uniparental disomy 7q; VAF, variant allele frequency; WGS, whole‐genome sequencing.

In contrast, three *SAMD9* variants were detected and classified as VUS in patients presenting with predominant neurodevelopmental features that are not classically associated with *SAMD9*‐related disease, including intellectual disability and autism spectrum disorder. In one case (P2), the coexistence of a previously reported pathogenic missense variant and a de novo truncating variant at similar VAF (54%) raises the hypothesis of a germline genetic rescue mechanism, potentially accounting for the absence of severe systemic manifestations.^17^

In another patient (P3), a de novo *SAMD9* missense variant was detected with a low VAF (26%); however, the clinical presentation was not clearly compatible with known *SAMD9*‐associated phenotypes. The absence of detectable UPD7q or other SGR mechanisms suggests possible post‐zygotic mosaicism, which may explain the lack of systemic involvement.

Finally, patient P4 carried a heterozygous missense variant that was also present in both asymptomatic parents, precluding determination of the transmitting parent. Genome‐wide analysis revealed multiple regions of homozygosity, suggesting possible parental relatedness or origin from a population with a higher rate of consanguinity, despite no reported consanguinity in the clinical history. No evidence of SGR was identified in either the patient or her parents. This variant has previously been reported in a patient with myelodysplastic syndrome but showed incomplete penetrance, as the mother and two siblings were unaffected carriers.^18^ Based on these observations, we suggest reclassifying this variant as a VUS.

### Identification of a previously unrecognized UPD7q somatic rescue mechanism leading to reclassification of a *SAMD9L* variant

**FIGURE 5:**
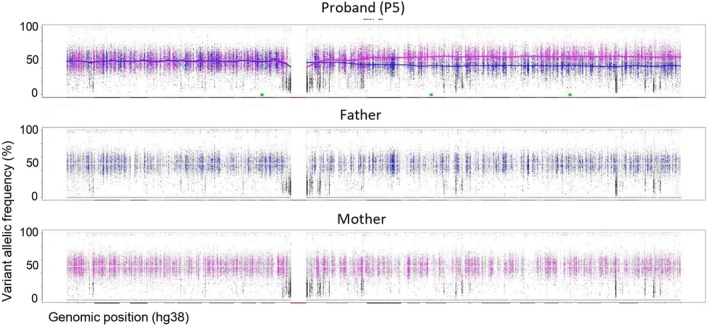
Low‐level mosaic sUPD7q discovered by SNP‐array‐like approach Whole‐genome SNP array analysis (Baracuda tool) leading to the reclassification of a *SAMD9L* variant identified in patient P5. Representation of all chromosome 7 variants in an SNP‐array–like plot. Blue indicates variants inherited from the father, pink indicates variants inherited from the mother and grey indicates non‐inherited variants. Blue and pink shifts in allelic ratios along chromosome arms 7p and 7q in patient P5 reveal clear mosaic segmental uniparental disomy (sUPD) of chromosome 7q. [Colour figure can be viewed at wileyonlinelibrary.com]

For *SAMD9L*, one patient (P5) exhibited a highly compatible phenotype combining transient childhood pancytopenia and progressive neurological involvement, associated with a de novo *SAMD9L* variant (NM_152703.5: c.4648T>C; p.Ser1550Pro) (Table 1). At the time of initial genome analysis, this variant was classified as a VUS due to insufficient evidence of pathogenicity, despite strong phenotypic concordance. The spontaneous resolution of pancytopenia suggested the presence of an SGR mechanism. Subsequent analysis using the novel mosaic UPD detection tool Baracuda, which was not available at the time of first analysis, revealed a clear mosaic sUPD7q, despite an apparently normal VAF of 46.6% (14/30 reads) (Figure 5). To further explain the spontaneous remission despite low mosaicism sUPD7q, we look for additional SGR mechanisms in *SAMD9L*. This patient also carries another variant in *SAMD9L* (NM_152703.5: c.2566_2569del, p.Ala856Ter) predicted to be LOF and with a VAF of 15% (5/33 reads). These findings allowed reclassification of the variant as likely pathogenic. The later discovery of another patient carrying the same variant reinforced this diagnosis.^19^

Conversely, a second previously reported *SAMD9L* variant (NM_152703.5: c.2807C>T; p.Thr936Ile) was identified in a 60‐year‐old adult patient (P6) with long‐standing hereditary spastic paraplegia and no haematological abnormalities, suggesting reduced penetrance, an atypical neurological presentation or an incidental finding unrelated to the primary phenotype.^20^ Notably, this variant was previously reported in a paediatric patient with myelodysplastic syndrome associated with monosomy 7, in whom a somatic truncating mutation in SAMD9L was also identified, consistent with a somatic genetic rescue mechanism. This discrepancy further highlights the variable expressivity and context‐dependent impact of SAMD9L variants.

Finally, we report a child with intellectual disability in whom two maternally inherited *SAMD9L* variants were identified (P7). The first variant (NM_152703.5: c.2519T>C; p.Met840Thr) affects the same residue as a previously reported pathogenic variant (p.Met840Lys),^19^ although it involves a less disruptive biochemical substitution. The potential contribution of the second missense variant (NM_152703.5: c.4231C>G; p.Arg1411Gly) in *cis* cannot be excluded and may represent a modifying effect or a germline rescue mechanism if associated with partial LOF.

## DISCUSSION

In this study, we combined a detailed case report describing a novel *SAMD9L* variant with a retrospective and transversal genome‐wide analysis of *SAMD9* and *SAMD9L* variants identified across a large rare disease sequencing cohort. This approach highlights both the expanding phenotypic spectrum associated with these genes and the substantial challenges inherent to variant interpretation in the context of highly dynamic genomic disorders characterized by SGR.

Consistent with previous reports, the majority of germline *SAMD9* and *SAMD9L* variants identified in our analysis were novel or extremely rare, with most being unique to a single individual or family. This observation mirrors published data showing that most *SAMD9/9L* variants are singletons.^9^ Consequently, a large proportion of variants remain classified as VUS, reflecting the limited availability of robust genotype–phenotype correlations and functional validation.

Variable expressivity and incomplete penetrance complicate the interpretation of SAMD9/SAMD9L variants, with clinical outcomes ranging from severe multisystem disease to apparently unaffected carriers, even within the same family. These findings underscore that the presence of a rare coding variant alone is insufficient to establish causality without careful phenotypic correlation and longitudinal follow‐up.

SGR represents a defining feature of *SAMD9/9L*‐associated disorders and plays a central role in shaping disease expressivity over time. Published data indicate that rescue mechanisms are detected in more than half of reported patients, with notable differences between *SAMD9* and *SAMD9L*.^12^ UPD7q, in particular, is significantly more frequent in *SAMD9L* mutation carriers. In our study, the identification of mosaic UPD7q in a patient with a compatible *SAMD9L* phenotype despite a normal VAF reinforces the importance of systematically searching for rescue mechanisms when interpreting germline variants. This example further underscores the need to integrate genetic rescue mechanisms into variant interpretation. In this context, the systematic analysis of both peripheral blood and a non‐haematopoietic tissue is crucial to distinguish true germline variants from haematopoietic‐restricted events and to accurately identify SGR that may mask the constitutional genotype. In addition, we report a patient harbouring two de novo, likely germline variants, one previously associated with a presumed GOF mechanism and the other classified as predicted LOF, raising the possibility of germline rescue and further complicating the interpretation of such variant combinations.

An important unresolved question concerns the predictability of SGR and its clinical consequences. Although rescue mechanisms such as monosomy 7, UPD7q or secondary LOF variants are frequently observed, their timing, tissue distribution and long‐term stability remain largely unpredictable. Moreover, the occurrence of cytopenias does not always strictly parallel the emergence of a detectable rescue event, suggesting that additional triggers, such as infectious stress, inflammatory activation or other environmental factors, may precipitate haematological decompensation in genetically predisposed individuals. Conversely, the presence of SGR does not invariably translate into durable haematological correction, as certain SGR events (e.g. monosomy 7) may themselves confer a risk of clonal progression.

Age‐dependent risk represents another critical aspect of *SAMD9/9L*‐associated disorders. The highest risk of severe haematological complications occurs during childhood, whereas adults may remain asymptomatic or develop milder, slowly progressive manifestations. In mice, heterozygous and homozygous *SAMD9L* knockout leads to late‐onset myelodysplasia in a subset of animals, a phenotype that is markedly exacerbated by infectious stress.^3^ These findings may explain why infections often precede cytopenias and monosomy 7 in young children with *SAMD9/9L* mutations, while neurological manifestations tend to emerge later and progress gradually during adolescence or adulthood. In line with this model, the index patient described in this report was initially classified as having an IEI prior to the subsequent development of pancytopenia. At the other end of the spectrum, the presence of rare *SAMD9/SAMD9L* variants in older individuals with limited clinical manifestations should be interpreted in light of the possibility of unrecognized haematological features during infancy, which may have resolved spontaneously and are frequently associated with SGR events.

Taken together, our results emphasize that rare coding variants in *SAMD9* and *SAMD9L* can be identified across a broad range of clinical indications in large‐scale sequencing programmes. However, only a subset of these variants demonstrates a convincing genotype–phenotype correlation consistent with known disease mechanisms. Careful integration of inheritance patterns, allelic balance, prior reports and evidence of somatic or germline genetic rescue is essential to avoid overinterpretation, particularly in patients lacking the cardinal haematological or multisystem features of *SAMD9/9L*‐associated disorders. Longitudinal genomic monitoring may help refine variant interpretation over time. Beyond individual cases, genome‐wide sequencing combined with large national datasets helps contextualize ultra‐rare variants across heterogeneous phenotypes and improves interpretation of SAMD9/SAMD9L‐associated disorders.

## AUTHOR CONTRIBUTIONS

**Laetitia Gouas:** Validation; formal analysis; writing – review and editing. **Mathieu Fusaro:** Writing – review and editing; writing – original draft; conceptualization; validation; formal analysis; supervision; investigation. **Virginie Bernard:** Software; resources; writing – review and editing. **Lise Larcher:** Writing – review and editing; validation; formal analysis. **AURAGEN Consortium:** Project administration; methodology; writing – review and editing; software. **Alexis Praga:** Software; resources; writing – review and editing. **Maud Tusseau:** Validation; formal analysis; writing – review and editing. **Marie Passet:** Validation; formal analysis; writing – review and editing. **Tristan Celse:** Validation; writing – review and editing; formal analysis. **Roman Klifa:** Writing – review and editing; resources; data curation. **Hadjer Dellal:** Formal analysis; writing – original draft; writing – review and editing.

## FUNDING INFORMATION

This research received no specific grant.

## CONFLICT OF INTEREST STATEMENT

The authors have no conflicts of interest to disclose.

## ETHICS STATEMENT

The study was conducted in accordance with the Declaration of Helsinki. All patients included in this study, or their parents in the case of children, gave informed consent for genetic studies. As it was a non‐interventional retrospective study (MR‐004), no declaration to data protection authority and no ethics committee approval were requested (JOR10‐10/05/2017).

## PERMISSION TO REPRODUCE MATERIAL FROM OTHER SOURCES

No previously published material has been reproduced. All figures, tables and graphical representations were generated by the authors.

## Supporting information


Data S1.

**Figure S1.** Immune phenotype of the proband. Proband measurements are denoted by red circles. Grey boxes represent age‐related reference ranges.
**Figure S2.** Read depth analysis across chromosome 7. Coverage profile across a large region of chromosome 7 (chr7:88257367–138 062176) encompassing the segment with altered allelic frequencies in the mother's FNA. The absence of any reduction in read depth argues against a mosaic interstitial deletion and supports a copy‐neutral event consistent with uniparental disomy of chromosome 7 (UPD7).
**Figures S3 and S4.** Mutational landscape of germline SAMD9/SAMD9L mutations visualized using ProteinPaint. Each circle represents an individual patient. Data are based on cases from Sahoo et al. (2025) and the PFMG2025 rare disease sequencing programme.

**Table S1.** Immune explorations.
**Table S2.** Complete blood count revealed pancytopenia in peripheral blood.

## Data Availability

Data are available upon request.
