# Supplementary material for: From a novel pathogenic SAMD9L variant to cohort‐wide insights: Whole‐genome sequencing highlights somatic genetic rescue and phenotypic heterogeneity
Source: Br J Haematol. 2026 May 19;209(1):75–83. doi: 10.1111/bjh.70563 (PMC13340485; doi:10.1111/bjh.70563)
Supplement: Supplementary file 1 — Data S1. [file BJH-209-75-s002.docx]

**Case report**

A 5-month-old full-term infant presented with poor weight gain and atopic dermatitis and was initially treated for suspected cow’s milk protein allergy and gastro-oesophageal reflux. She subsequently developed feeding refusal followed by a hypotonic cyanotic episode and was admitted for respiratory distress requiring oxygen therapy. Bronchoalveolar lavage confirmed Pneumocystis jirovecii and CMV infection, with evidence of systemic CMV replication. HIV testing was negative. Treatment with cotrimoxazole and intravenous ganciclovir led to gradual respiratory improvement.

Immunological investigations showed normal immunoglobulin levels and preserved vaccine responses. However, lymphocyte proliferation was reduced upon OKT3 stimulation. Flow cytometry revealed B-cell and NK-cell lymphopenia with a normal T-cell phenotype (Supplemental Figure 1 and Supplemental Table 1). Altogether, these findings were suggestive of an underlying cellular immunodeficiency.

A few weeks later, laboratory investigations revealed pancytopenia on complete blood count (Supplementary Table 2). Bone marrow aspiration showed a normocellular marrow with erythroid hyperplasia. Rare granulocytic and erythroid precursors displayed cytoplasmic vacuoles. Mild dysmegakaryopoiesis was observed, with approximately 20% of megakaryocytes showing hypolobation or karyorrhexis, along with very mild late-stage dyserythropoiesis. No abnormal infiltrate was identified. Differential diagnoses included drug toxicity (notably ganciclovir-related) and early myelodysplastic syndrome, without definitive conclusion.

Cytogenetic analysis showed no clonal abnormalities, and FISH analysis did not detect deletion of chromosome 7q or monosomy 7. A targeted next-generation sequencing panel covering 62 genes associated with myeloid malignancies and inherited predisposition to myelodysplastic syndromes identified a *SAMD9L* variant (NM_152703.5: c.4529G>C; p.Gly1510Ala) with a variant allele frequency (VAF) of 24%. Given this relatively low VAF, a somatic versus germline origin could not be determined at this stage and required further investigation using non-hematopoietic tissue and familial genetic studies. The variant was initially classified as a variant of uncertain significance (VUS).

**Supplementary Methods**

**Baracuda tool**

The relative excess of variants inherited from one parent was estimated using trio WGS data according to the Auragen specification. Using variants called in the index case with sufficient coverage, parental inheritance was inferred using Mendelian segregation rules. A Wilcoxon rank-sum test was implemented to detect aneuploidies, copy-number variants (CNVs), and uniparental disomies in both mosaic and non-mosaic states.

For each relevant case, the B-allele frequency (BAF) was plotted and color-coded by parental origin. To facilitate detection of low-level mosaicism, average parental contribution was computed using a sliding window approach and overlaid on the same plot. Because BAF plots alone cannot distinguish between aneuploidies, CNVs, and disomies, read depth across all chromosomes and copy number estimates for each parent were also visualized.

**NGS panel sequencing**

The myeloid neoplasia gene panel include a minimal set of the following 108 genes : *ABL1, ARID2, ASXL1, ASXL2, ASXL3, BAK1, BAX, BCL2, BCL2L1/BCLXL, BCOR, BCORL1, BRAF, BRCC3, CALR, CBFB, CBL, CCND1, CCND2, CEBPA, CHEK2, CREBBP, CSF3R, CSNK1A1, CTCF, CUX1, DDX41, DHX15, DNM2, DNMT3A, EBF1, EED, EIF6, EP300, ETNK1, ETV6, EZH2, FLT3, GATA2, GNAS, GNB1, H3-3A/H3F3A, HRAS, ID3, IDH1, IDH2, IRF1, JAK1, JAK2, JAK3, KDM6A, KIT, KLC2, KMT2A, KMT2D, KRAS, LUC7L2, MAP2K1, MCL1, MECOM, MEN1, MGA, MPL, MYC, NF1, NFE2, NPM1, NRAS, PDGFRA, PDS5B, PHF6, PHIP, PIGA, PPM1D, PRPF8, PTEN, PTPN11, RAD21, RUNX1, SAMD9, SAMD9L, SETBP1, SETD1B, SETD2, SF1, SF3B1, SH2B3, SMC1A, SMC3, SRSF2, STAG2, STAT3, STAT5B, SUZ12, TCF4, TERC, TERT, TET2, TP53, TRIB1, U2AF1, U2AF2, UBA1, UBE2A, UBTF, WT1, ZBTB33, ZBTB7A, ZEB2, ZNF384, ZRSR2*.

**Variant filtering strategy for database transversal analysis**

For this transversal analysis, we applied stringent filtering criteria to retain variants with an allelic balance ≥25%, compatible with heterozygous or homozygous germline variants, and excluded low-level somatic events. Only rare variants were considered, defined by an allele frequency <1% both in the internal cohort and in population databases, with a gnomAD allele count <50. Variants were further restricted to those affecting the coding sequence or canonical splice sites.

Subsequent prioritization focused on variants previously reported in affected individuals or occurring de novo, when parental data were available. Variant interpretation integrated population frequency, in silico prediction tools, conservation data, and published literature. For each individual, genomic data were additionally reviewed to identify potential mechanisms of somatic or germline genetic rescue, including copy-number alterations, UPD7q and secondary loss-of-function variants. Clinical information was extracted from standardized phenotypic annotations to assess concordance with known SAMD9- or SAMD9L-associated disease spectra.
