# Supplementary material for: From a novel pathogenic SAMD9L variant to cohort‐wide insights: Whole‐genome sequencing highlights somatic genetic rescue and phenotypic heterogeneity
Source: Br J Haematol. 2026 May 19;209(1):75–83. doi: 10.1111/bjh.70563 (PMC13340485; doi:10.1111/bjh.70563)
Supplement: Supplementary file 2 — Figure S1. Immune phenotype of the proband. Proband measurements are denoted by red circles. Grey boxes represent age‐related reference ranges. Figure S2. Read depth analysis across chromosome 7. Coverage profile across a large region of chromosome 7 (chr7:88257367–138 062176) encompassing the segment with altered allelic frequencies in the mother's FNA. The absence of any reduction in read depth argues against a mosaic interstitial deletion and supports a copy‐neutral event consistent with uniparental disomy of chromosome 7 (UPD7). Figures S3 and S4. Mutational landscape of germline SAMD9/SAMD9L mutations visualized using ProteinPaint. Each circle represents an individual patient. Data are based on cases from Sahoo et al. (2025) and the PFMG2025 rare disease sequencing programme. [file BJH-209-75-s001.pptx]

## Slide 1
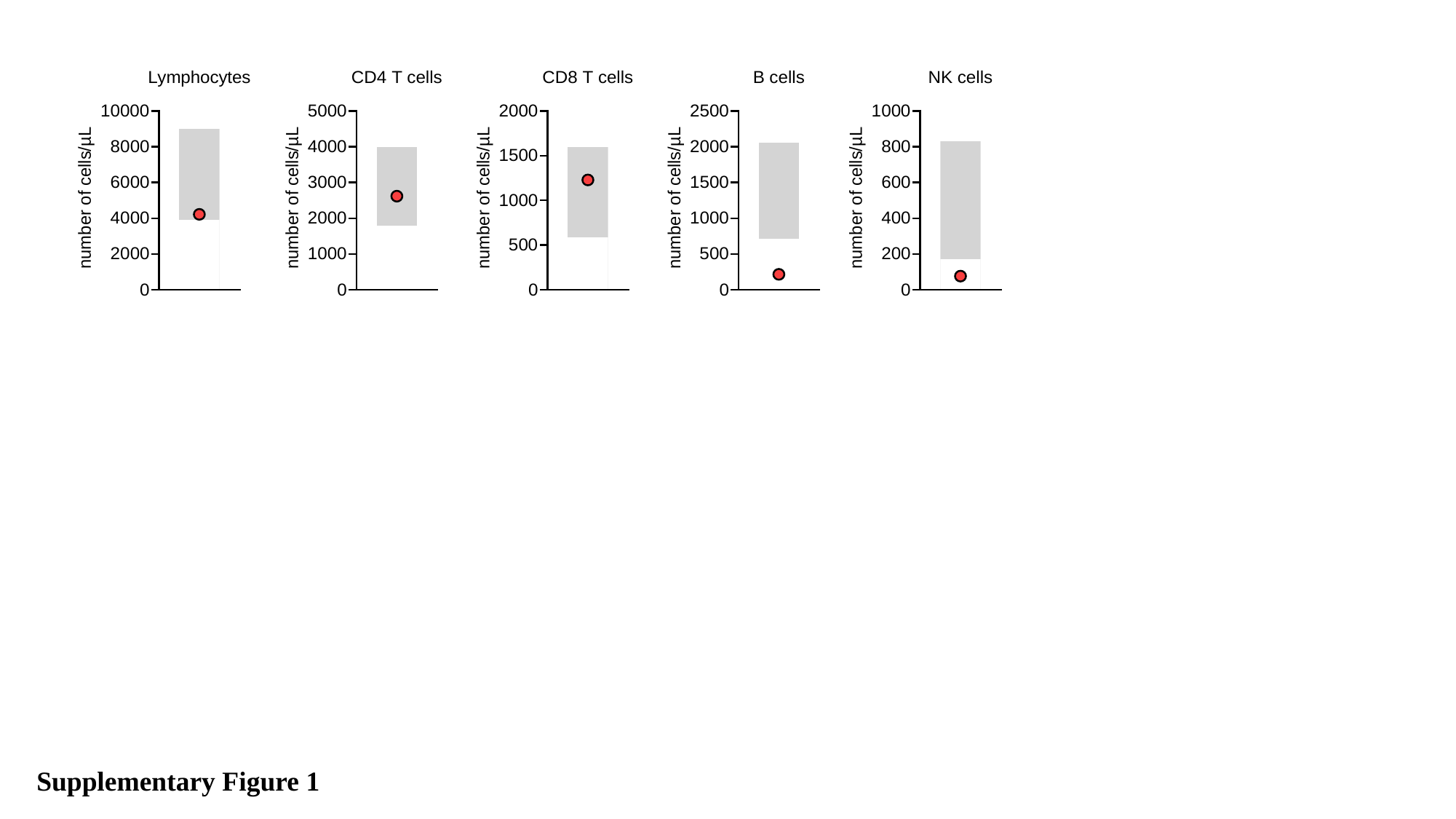

Supplementary Figure 1

## Slide 2
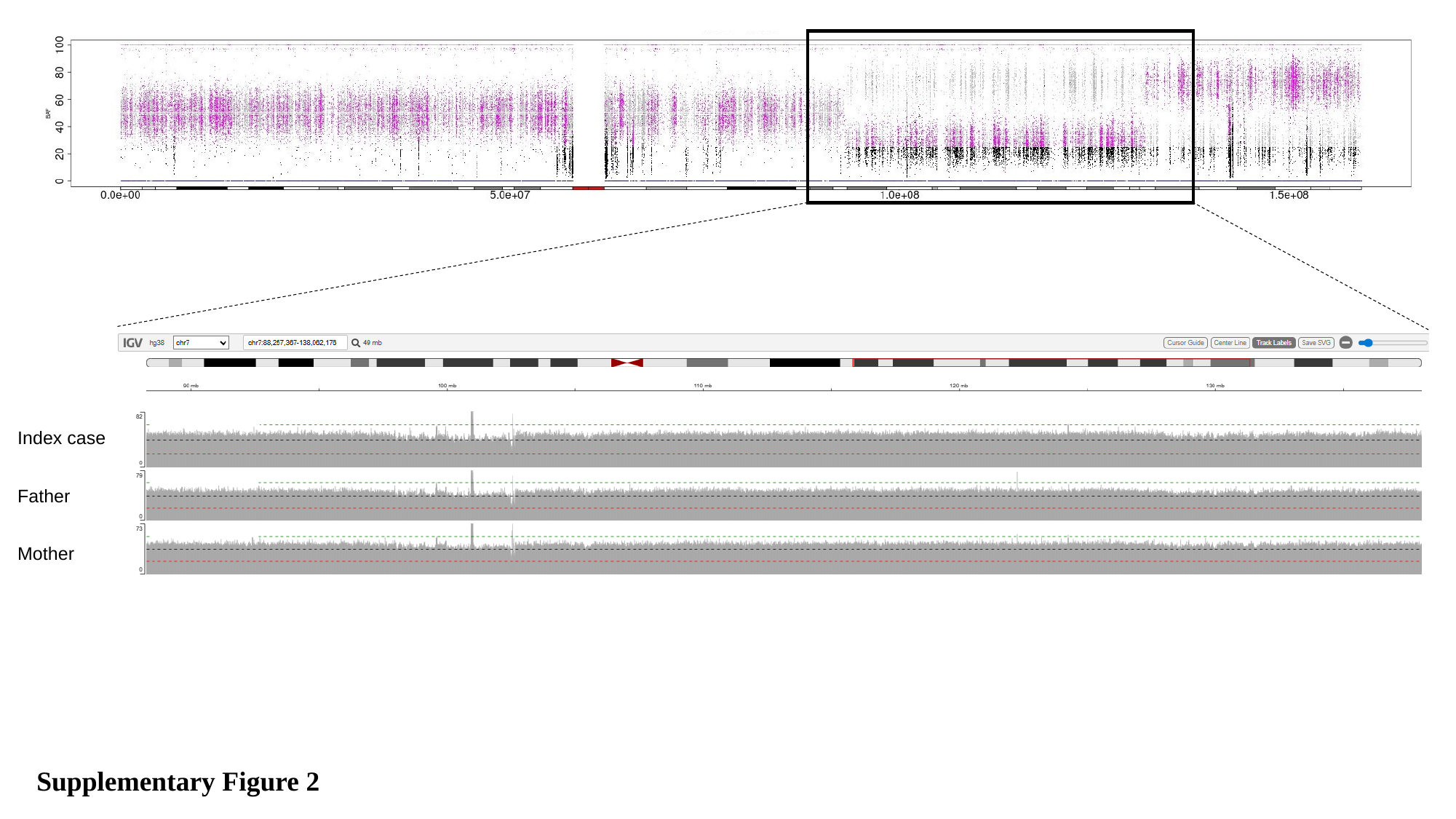

Index case
Father
Mother
Supplementary Figure 2

## Slide 3
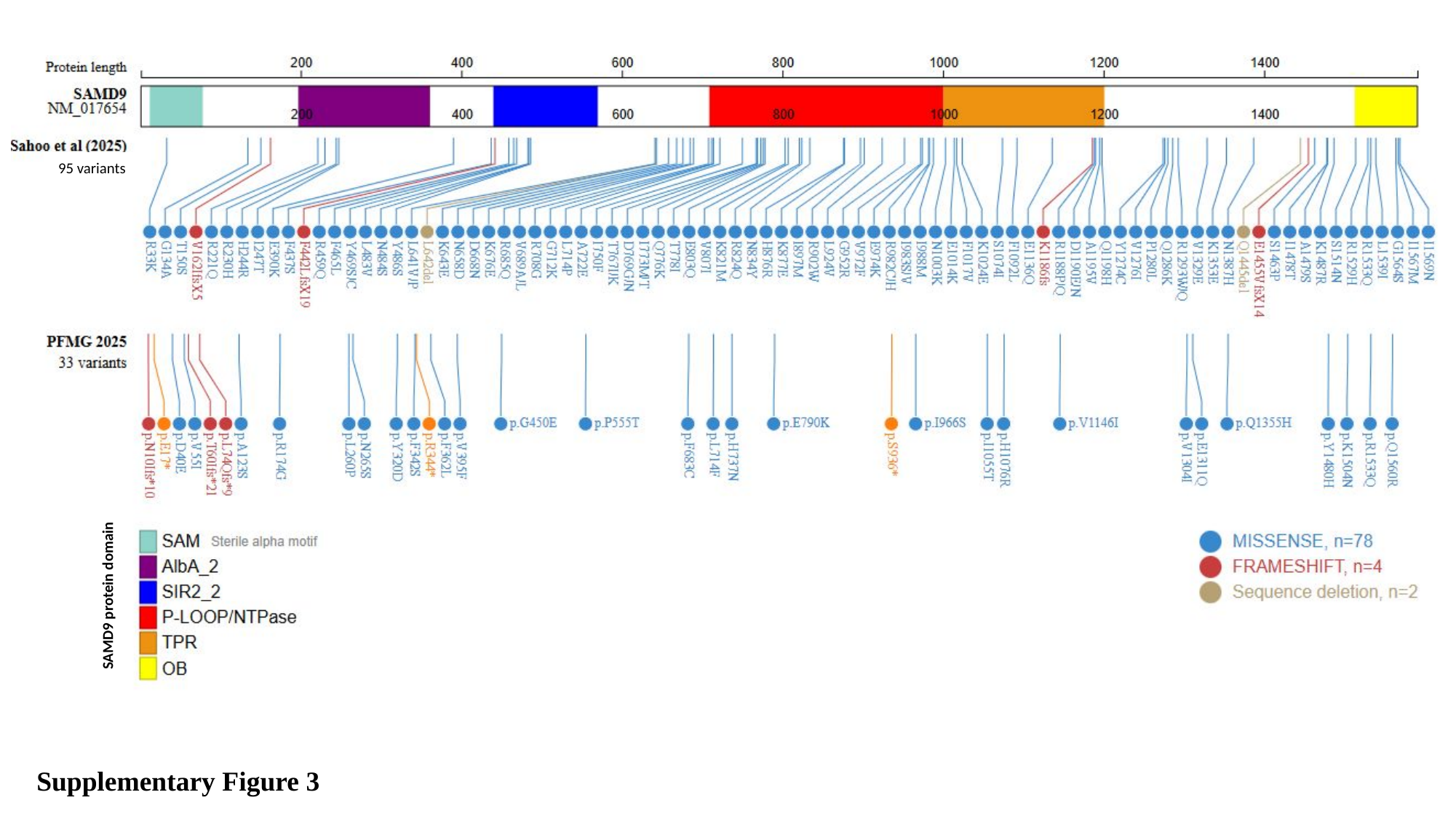

95 variants
SAMD9 protein domain
Supplementary Figure 3

## Slide 4
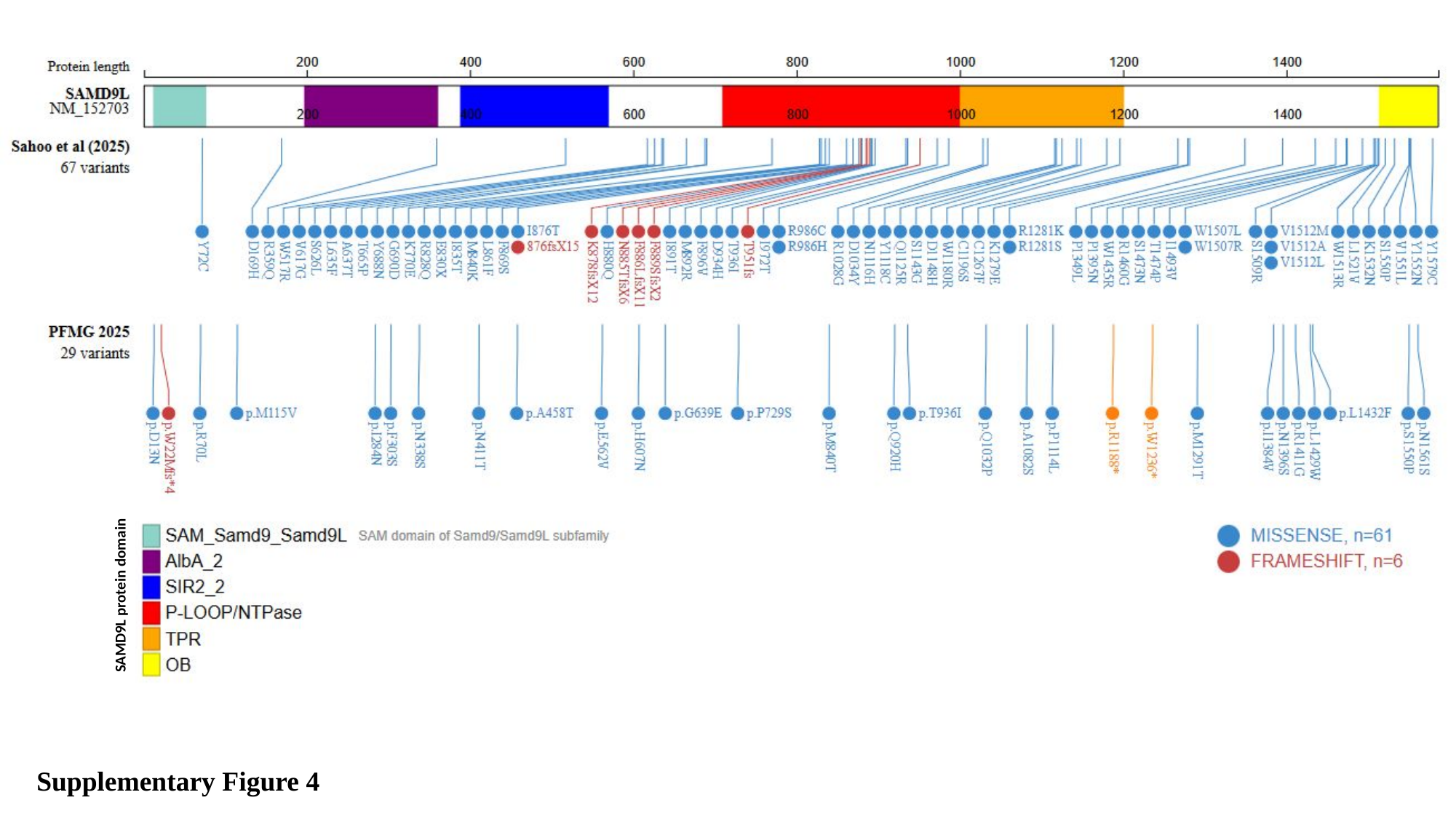

SAMD9L protein domain
Supplementary Figure 4
