# Supplementary material for: From a novel pathogenic SAMD9L variant to cohort‐wide insights: Whole‐genome sequencing highlights somatic genetic rescue and phenotypic heterogeneity
Source: Br J Haematol. 2026 May 19;209(1):75–83. doi: 10.1111/bjh.70563 (PMC13340485; doi:10.1111/bjh.70563)
Supplement: Supplementary file 3 — Table S1. Immune explorations. Table S2. Complete blood count revealed pancytopenia in peripheral blood. [file BJH-209-75-s003.docx]

**Supplemental Table 1. Immune explorations**

|  | **Patient** | **Normal range** |
| --- | --- | --- |
| **Absolute count** |  |  |
| Lymphocytes (cells/µL) | 4323 | 3900-9000 |
| CD3 (cells/µL) | 4012 | 2500-5600 |
| CD3+CD4+ (cells/µL) | 2475 | 1800-4000 |
| CD3+CD8+ (cells/µL) | 1392 | 590-1600 |
| CD19+ (cells/µL) | **185** | 712-2059 |
| CD56+ (cells/µL) | **117** | 170-830 |
|  |  |  |
| **T cell phenotype** |  |  |
| RTE CD4 (%/CD3+CD4+) | **77.6** | 60-72 |
| CD45RA+ CD4 (%/CD3+CD4+) | 92 | 88-95 |
| CD45RO+ CD4 (%/CD3+CD4+) | 8 | 6-23 |
| Naive CD8 (%/CD3+CD8+) | 79.8 | 75-94 |
| CM CD8 (%/CD3+CD8+) | 1.3 | 0.5-6 |
| EM CD8 (%/CD3+CD8+) | 8.4 | 0.5-10 |
| EMRA CD8 (%/CD3+CD8+) | 10.4 | 1-11 |
| MAIT (%/CD3+) | 0.1 | 0.1-0.5 |
| Va7.2 (%/CD3+) | 2.5 | 1.2-2.8 |
|  |  |  |
| **Serum Ig** |  |  |
| IgG (g/L) | **4,24** | 2.03-9.48 |
| IgA (g/L) | 0,11 | 0.08-0.91 |
| IgM (g/L) | **0,29** | 0.17-1.50 |
|  |  |  |
| **T cell proliferation** |  |  |
| PHA (%) | 93.6 | *> 60* |
| OKT3 (%) | **18** | *> 40* |

**Supplemental Table 2. Complete blood count revealed pancytopenia in peripheral blood.**

|  | Patient | Normal range |
| --- | --- | --- |
| Leukocytes (G/L) | 3.98 | 3.93- 16.87 |
| Red blood cells (RBCs) (T/L) | **2.5** | 4.07-5.25 |
| Hemoglobin (g/dL) | **7.6** | 10.2-13.8 |
| Hematocrit (%) | **21.8** | 30.7-40.1 |
| Mean corpuscular volume (MCV) (fL) | **87.2** | 69.8-82.4 |
| Mean corpuscular hemoglobin (MCH) (pg) | **30.4** | 23.0-28.4 |
| Mean corpuscular hemoglobin concentration (MCHC) (g/dL) | 34.9 | 31.7-36.1 |
| Red cell distribution width (RDW) (%) | 16.4 | 11.5- 14.5 |
| Platelets (G/L) | **34** | 208-486 |
| Immature platelet fraction (IPF) (%) | 4.10% | 1.2-8.9 |
| Reticulocytes (G/L) | 77 | 20-80 |
| Neutrophils (G/L) | **0.46 (11.6 %)** | 1.50 - 6.53 |
| Eosinophils (G/L) | 0.13 (3.3 %) | 0.00 - 0.88 |
| Basophils (G/L) | 0.00 (0 %) | 0.00 - 0.14 |
| Lymphocytes (G/L) | 3.26 (81.8 %) | 2.08 - 10.32 |
| Monocytes (G/L) | 0.13 (3.3 %) | 0.00 - 1.55 |
